# Tracking Red Palm Mite Damage in the Western Hemisphere Invasion with Landsat Remote Sensing Data

**DOI:** 10.3390/insects11090627

**Published:** 2020-09-11

**Authors:** Jose Carlos Verle Rodrigues, Michael H. Cosh, E. Raymond Hunt, Gilberto J. de Moraes, Geovanny Barroso, William A. White, Ronald Ochoa

**Affiliations:** 1 Center for Excellence in Quarantine and Invasive Species, University of Puerto Rico (UPR), San Juan, PR 00926, USA; jose_carlos@mac.com; 2 Hydrology and Remote Sensing Laboratory, Bldg. 007, Rm. 104, BARC-West, USDA-ARS, Beltsville, MD 20705, USA; raymond.hunt@usda.gov (E.R.H.J.); Alex.White@usda.gov (W.A.W.); 3 Departamento de Entomologia e Acarologia, ESALQ-Universidade de São Paulo, Piracicaba 13418-900, São Paulo, Brazil; moraesg@usp.br (G.J.d.M.); geovannybarroso@usp.br (G.B.); 4 Systematic Entomology Laboratory, Bldg. 005, Rm. 137, BARC-West, USDA-ARS, Beltsville, MD 20705, USA; ron.ochoa@usda.gov

**Keywords:** *Raoiella indica* Hirst, leaf damage, coconut palm, *Cocos nucifera* L., Google Earth Engine, Landsat time series, GNDVI

## Abstract

**Simple Summary:**

The red palm mite is a destructive pest for palm trees, impacting their productivity. Detection of their presence is important for management and the prevention of spread. Remote sensing may provide an opportunity to monitor and detect red palm mite presence using readily available land surface remote sensing, such as the Landsat satellite constellation. A study was conducted to determine if Landsat products are able to detect infestations at select sites in the Caribbean, Central, and South America. After a time series analysis, we determined that there are several impediments to detecting red palm mite damage at palm plantations.

**Abstract:**

Red palm mites (*Raoiella indica* Hirst, Acari: Tenuipalpidae) were first observed in the western hemisphere on the islands and countries surrounding the Caribbean Sea, infesting the coconut palm (*Cocos nucifera* L.). Detection of invasive pests usually relies upon changes in vegetation properties as result of the pest activity. These changes may be visible in time series of satellite data records, such as Landsat satellites, which have been available with a 16-day repeat cycle at a spatial resolution of 30 m since 1982. Typical red palm mite infestations result in the yellowing of the lower leaves of the palm crown; remote sensing model simulations have indicated that this feature may be better detected using the green normalized difference vegetation index (GNDVI). Using the Google Earth Engine programming environment, a time series of Landsat 5 Thematic Mapper, Landsat 7 Enhanced Thematic Mapper Plus and Landsat 8 Operational Land Imager data was generated for plantations in northern and northeast Brazil, El Salvador, and Trinidad-Tobago. Considering the available studied plantations, there were little or no differences of GNDVI before and after the dates when red palm mites were first revealed at each location. A discussion of possible alternative approaches are discussed related to the limitations of the current satellite platforms.

## 1. Introduction

Invasive species can be devastating to an ecosystem, and especially disruptive to an agro-ecosystem, which is not naturally sustainable without human intervention. *Raoiella indica* Hirst [1], known as the red palm mite (RPM), is one such invasive species. Plant hosts for RPM are mostly in the Arecaceae (palm family), with the coconut palm (*Cocos nucifera* L.) being economically important in the Caribbean region [2]. In Brazil, coconut production has importance in the eastern region of the state of Amazonas, and some states of the northeastern region [3]. The invasive *Raoiella* mites negatively impact coconut industries with losses of more than 70% of the production and death of new plants [4].

Conventional detection of RPM is very laborious and time consuming, especially when population densities need to be estimated [5]. Because RPM affects commodity prices, dissemination of infestation locations and onset dates are sometimes withheld to mitigate economic impacts. Remote sensing may provide a method of determining when and where an infestation occurred [6,7,8]. Furthermore, remote sensing may provide an estimate of RPM population densities and economic impact based on the amount of damaged foliage.

During the spread of RPM through the Caribbean region, there has been significant remote sensing coverage in place, with several satellite platforms that provide multispectral data in the visible, near infrared (NIR), and the shortwave infrared (SWIR) ranges. Data products from the Landsat series of satellites are at a scale where it is feasible to observe RPM damage, at locations that cannot be confirmed via ground observation [9]. This study was initiated to assess the ability of the Landsat constellation to detect RPM impacts on coconut palm plantations at three locations by analyzing imagery before and after the approximate date of introduction.

## 2. Background

### 2.1. Taxonomy and Geography

The taxonomic status of the genus *Raoiella* is considered complicated because of its unconnected collecting history. From 1924 to the present, 22 species of *Raoiella* were described from around the world. Additionally, numerous species were subsequently described from India and Pakistan [10,11,12,13,14]. Most of the valid species described are from Australia, with three species from India (including *R. indica*), one species from South Africa, and one species from Greece [15]. The genus is divided into five species groups, with *R. indica* and *Raoiella pandanae* Mohanasundaram composing the *R. indica* group, which are also the only species known to feed on monocotyledon plants [15]. Recent studies have indicated that many of the described *Raoiella* species from the Indian subcontinent were actually junior synonyms of *R. indica* [15]. Based on molecular evidence, Dowling et al. [16,17] indicated a possible African origin of *Raoiella* because the two most basal clades were from the Middle East and South Africa. However, with the recent collections, the geographic origin of the genus is now thought to be Australia [15,16]. RPM was known only from countries in the Eastern Hemisphere affecting coconut, areca, and date palms [15,17]. It is most likely that India was the source population of RPM for the Western Hemisphere. RPM migrated south and west through Mauritius, Reunion Island, Madagascar, and Africa around 1942 [18]. During the last 10 years, there has been an explosion of new RPM associations numbering over 100 host species [19,20]. Further research is needed to determine the nature of these plant associations, whether they are transitory, accidental, or truly new hosts.

**Figure 1:**
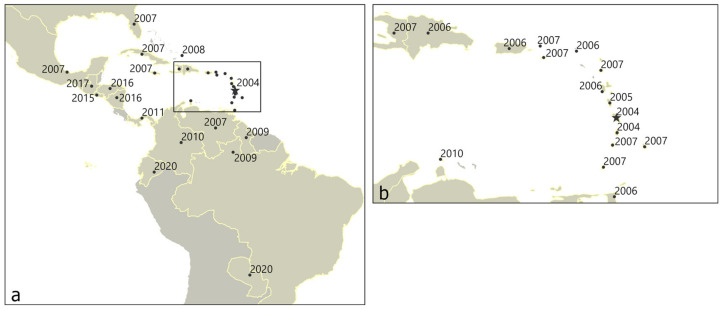
(**a**) Spread of the red palm mite (RPM, *Raoiella indica* Hirst) in the Western Hemisphere; (**b**) Island hopping in the Caribbean region. Locations indicate centroid of country, if no exact location of first observation is available. References in Table A1.

As a result of ocean debris, weather systems, or contamination from the harvesting of palm plants, the RPM spread across the Atlantic Ocean into the Western Hemisphere (Figure 1a, Table A1 in Appendix A). RPM were first observed in Martinique in 2004 and subsequent reports indicated a fast dispersal through the Antilles islands (Figure 1b) [4,16,21]. Later, Trinidad was invaded in 2006 [2] and El Salvador in 2015 [22]. The mite was first confirmed in 2009 to be infecting coconut palms in the Brazilian state of Roraima [23], and subsequently in the Amazonas state [24]. It later spread to other parts of the country [24,25,26,27]. RPM was reported in Ceará state in 2016 [27]. Trinidad, El Salvador, and Brazil were the three areas selected for the Landsat time series analysis. An additional site in Para, Brazil was also analyzed for comparison at a site with no observations of RPM.

**Figure 2:**
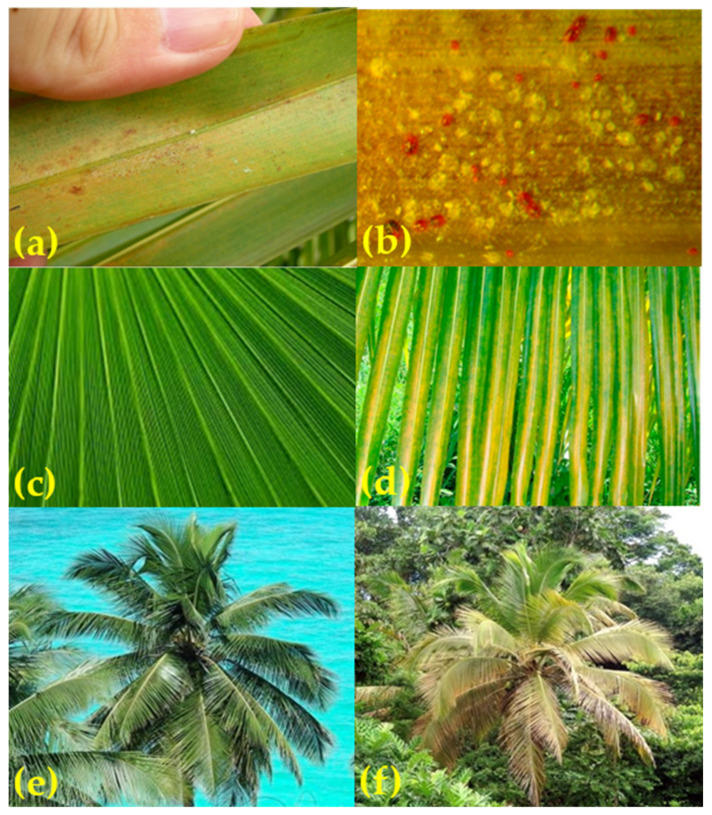
Effects of RPM on coconut palms. (**a**) Infestation on abaxial surface; (**b**) magnified abaxial surface showing damage; (**c**) healthy pinnae; (**d**) chlorotic pinnae; (**e**) healthy palm; (**f**) RPM-damaged palm.

The invasive dispersal behavior that has allowed RPM to spread worldwide has been linked to agricultural settings where the mite has become a pest. RPM mitigation was reduced in some areas by extensive spraying of trees with acaricides [28] and the establishment of natural enemies [20]. Damage by RPM infestation is manifested by the yellowing of the lower palm leaves as a result of RPM blocking closure of stomata, visible in high-spatial-resolution imagery (Figure 2), causing an increase in the transpiration and loss of water from the leaves. These leaves then begin to droop due to the water stress. Rodrigues et al. [29] described the symptoms of feeding damage by RPM in coconut plants appearing first as small pallid, yellow spots on the abaxial surface of the pinnae, where the mite infestation was located, later developing into larger, chlorotic spots (Figure 2). Dense colonies of mites are found feeding under the abaxial leaflet, primarily close to the leaflet midrib. The feeding damage causes the two sides of the leaflet to fold onto each other, with the mites remaining in the protected leaf fold. Considering the general appearance of the plant, as the feeding progresses, the bright green pinnae turns pale green, then yellow, and finally a copper-brown (Figure 2).

The spatial distribution of the mite within a palm tree was studied by Roda et al. [4], who reported that fronds located in the middle stratum of a palm hosted more mites than fronds from the upper or lower canopy and fronds from the lower stratum, on average, had fewer mites than the two other strata. This results from mite movement, feeding behavior, degradation of old feeding sites, and development of new fronds. Consequently, fronds located in the lower and middle strata are the ones primarily showing distinguishable feeding damage, which would increase the difficulty in satellite detection. High densities of damaged leaves near the top of the canopy are required for detection by nadir remote sensing in order to increase the spectral difference between healthy and damaged canopies.

### 2.2. Remote Sensing with Landsat Satellites

**Figure 3:**
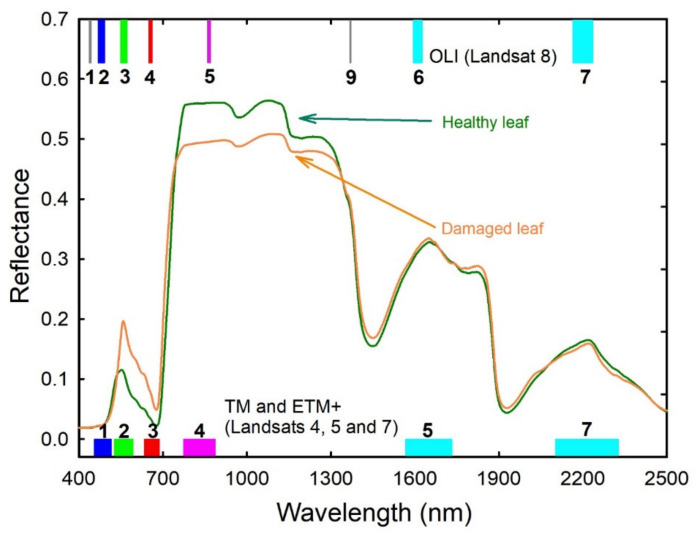
Simulated spectral reflectances of coconut palm leaves using the PROSPECT model. Along the bottom, sensor band number and wavelengths are shown for the Thematic Mapper (TM) and Enhanced Thematic Mapper Plus (ETM+) sensors, and along the top for the Operational Land Imager (OLI). Exact wavelengths are shown in Table A2. Because parameterization data for coconut palms were not available, these simulations were based on studies using healthy oil-palm leaves, along with leaves damaged by *G. boninensis*.

The Landsat 5 Thematic Mapper (L5-TM), Landsat 7 Enhanced Thematic Mapper Plus (L7-ETM+), and Landsat 8 Operational Land Imager (L8-OLI) have been providing 30-m resolution remote sensing data with a 16-day exact repeat cycle since 1984 (Table A2). The first Landsat satellite was launched in 1972 as the first civilian mission to monitor Earth’s resources from space [30]. At the time, aerial photography was the primary method for measuring defoliation by insects, but it was soon realized that digital data from satellites offered important benefits such as automated image processing and repeated coverage of large areas [31,32]. However, there was also an immediate demand for higher-spatial-resolution imagery with more spectral bands. In response to this demand, Landsat 4 and Landsat 5 were launched in 1982 and 1984, respectively, with the Thematic Mapper sensor that has 6 spectral bands in the visible, NIR, and SWIR ranges (Figure 3). The new bands allowed for better assessments of forest health [7,33,34].

Commercial satellites provide much higher spatial resolutions, at the expense of being unable to provide repeated global coverage at low cost. A higher-resolution panchromatic band was included on the upgraded L7-ETM + [30,35]. The importance of atmospheric correction of digital imagery to land-surface reflectance was shown by the success of NASA’s Moderate Resolution Imaging Spectroradiometer, so L8-OLI (Figure 3) included new spectral bands for detection of coastal aerosols and wispy cirrus clouds [36,37].

The data for 37 years of Landsat TM, ETM+, and OLI imagery (Table A2) for a given location may be gathered automatically to form a time series (called a data stack). Plantations and forest stands may exist for about 30–60 years, so the data stack may be used to show the specific historical changes that occurred over time, such as invasion by invasive mites [38,39]. The recent development of Google Earth Engine allows for rapid processing of Landsat data stacks [40]. For example, Lee et al. [41] used Google Earth Engine to detect and monitor changes in industrial oil palm plantations (*Elaeis guineensis* Jacq).

Spectral vegetation indices are useful for contrasting spectral wavelengths, as well as reducing problems with topography. The standard vegetation index is the normalized difference vegetation index (NDVI):

NDVI = (R_NIR_ − R_R_) / (R_NIR_ + R_R_) (1)

where R_NIR_ and R_R_ are the canopy reflectances for the near infrared and red wavebands [42,43]. A spectral index combining both green and NIR wavebands is the green normalized difference vegetation index (GNDVI):

GNDVI = (R_NIR_ − R_G_)/(R_NIR_ + R_G_) (2)

where R_G_ is the canopy reflectance for the green waveband [44].

## 3. Methods

### 3.1. PROSAIL Simulations

The question is what kind of changes are expected to occur in palm plantations invaded by *R. indica*. Coconut palm fronds infested with red spider mites are yellow, hold less water, and droop downward [29]. Leaf and canopy radiative transfer models use measured leaf data (chlorophyll, dry matter, and moisture contents), leaf area index, and leaf angle distributions to simulate spectral reflectances from a vegetation canopy. Simulations from the combined leaf and canopy model, PROSPECT and SAIL (Scattering by Arbitrary Inclined Leaves) [45,46] were used to make predictions on the spectral changes seen by remote sensing. However, we were unable to find studies in the peer-reviewed literature with leaf and spectral data of healthy and RPM-infested coconut palms.

The observed symptoms of damaged *C. nucifera pinnae* are very similar to those of oil palms (*Elaeis guineensis Jacq*.) infected with basal stem rot (*Ganoderma boninensis*), and there are many studies which are suitable for PROSAIL (combination of PROSPECT and SAIL) model parameterization [47,48,49,50,51]. Total chlorophyll and moisture contents for healthy oil palm pinnae were obtained from Gapor et al. [52], leaflet dry matter contents were obtained from Awal et al. [53], leaf area index from Tan et al. [54], and leaf angle distributions from Lelong et al. [47] and Ahmadi et al. [51]. The simulated spectral reflectances of healthy and damaged coconut palm canopies were converted to band reflectances using the published relative response functions for the L5-TM, L7-ETM + and L8-OLI sensors [55].

### 3.2. Change Detection Analysis

A change detection algorithm was employed to detect any significant changes in the time series of GNDVI using a Python library, Changefinder (version 0.03, https://pypi.org/project/changefinder/, downloaded on 26 August 2020). The basis of this analysis is sequentially discounted autoregressive (SDAR) time series modeling [56,57]. This type of analysis has found several uses in data science, including network security analysis [56] and electroencephalogram analysis [58]. This program processes a time series and produces a temporal anomaly score, which identifies significant changes in a time series, as evidenced in a large increase in the score. These scores are plotted along with the GNDVI series in the subsequent plots. For the beginning of the time series, the anomaly score can be large, because the window of analysis still includes a non-existent time signal. After the temporal analysis is sufficiently within the time series, the anomaly score becomes stable and deviations are then measured from this stable score. Any large (relative to the variability of the signal) anomaly indicates a change in the temporal pattern of the signal. An anomaly plot has an initial large value as a result of this “spinup”. Subsequent large anomalies indicate alterations in the time series.

### 3.3. Study Sites

Four study sites located in El Salvador, Trinidad, and central and northern Brazil (Table 1) were selected for this analysis because of their large area of palms (>100 m × 100 m) and the direct observation by the authors of the presence of *R. indica* in three of these locations. The typical infestation observed by the authors was large in scale, with every tree in the domain exhibiting significant *R. Indica* impact, as previously observed in [5]. Therefore, it is assumed that within the study sites, the infestation is total. A key issue with analyzing satellite data everywhere on Earth, but especially in the tropics, is the presence of clouds, which have dramatic impact on any quantitative calculation without effective cloud masking. Furthermore, given the size of the clouds (small scale cumulus from convective development), small regions of interest were designated for analysis and screened for being ‘cloud-free’. Large regions of interest would have less frequent cloud-free instances, so to maximize the number of retrieved scenes, small domains were preferred, but still consisting of at least 100 pixels of 30 by 30 m.

## 4. Results

### 4.1. Predicted Remote Sensing Signal for RPM damage

PROSAIL model simulations indicate that canopy spectral reflectances in the NIR (TM band 4 or OLI band 5) should be greater in healthy palm plantations and reflectances in the green (TM band 2 or OLI band 3) should be greater for infected palm plantations (Figure 3).

**Figure 4:**
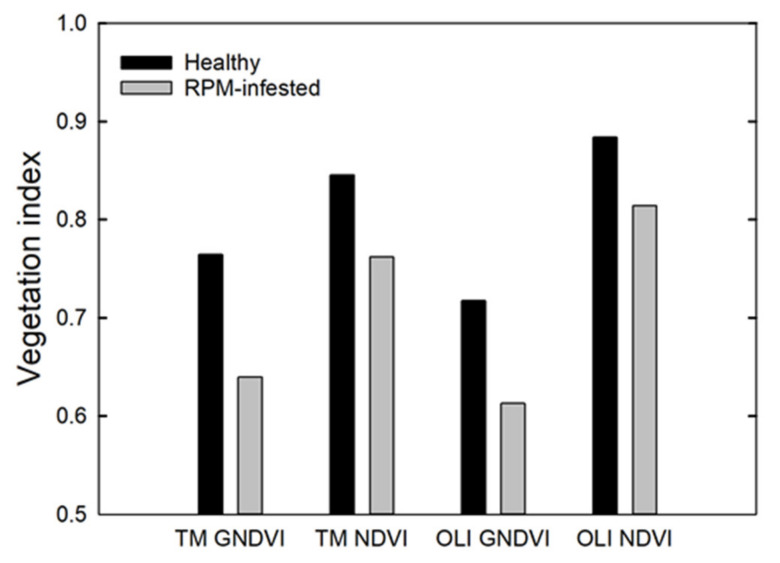
Predicted Green Normalized Difference Vegetation Index (GNDVI) and Normalized Difference Vegetation Index (NDVI) for Landsat 5-Thematic Mapper (L5-TM) and Landsat 8 Operational Land Imager (L8-OLI) based on simulated canopy reflectance spectra of healthy and damaged coconut palms simulated using the PROSPECT and SAIL model.

GNDVI shows larger differences between healthy and damaged palm trees compared to NDVI (Figure 4). Because of a small shift in wavelengths (Table A2), GNDVI slightly decreases and NDVI slightly increases when the operational sensors changed from L5-TM to L8-OLI (Figure 4). Therefore, we calculated GNDVI for the Landsat time series for each study site.

### 4.2. Trinidad Study Site

**Figure 5:**
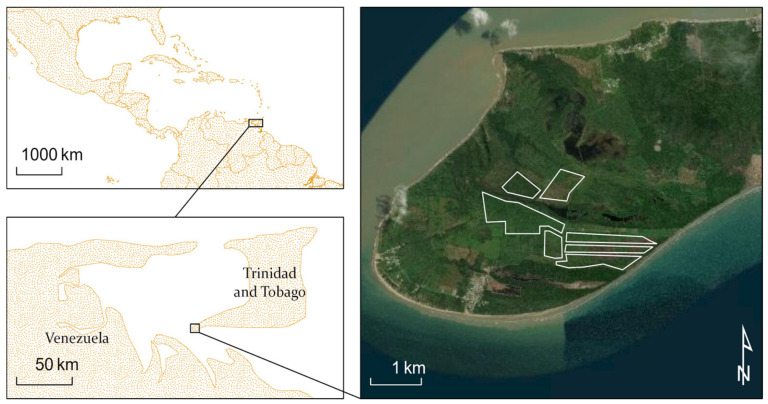
Coconut palm plantation in Trinidad shown in a true color Google Earth image. The area outlined in white was selected for data retrieval from Google Earth Engine.

**Figure 6:**
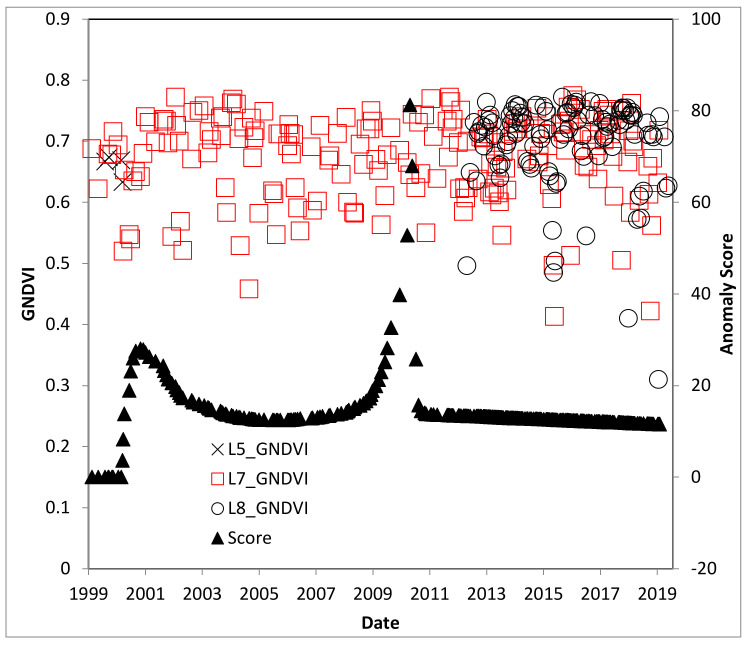
GNDVI time series for the Trinidad study site, with L5-TM prior to 2013, L8-OLI after 2013, and Landsat 7 Enhanced Thematic Mapper (L7-ETM+) for the entire period. *R. indica* was first observed in 2006.

Figure 5 shows the selected palm stands for the study with true color. A significant portion of the L5-TM scenes for southern Trinidad were not used because of extensive cloud cover, but there were significantly better results for L7-ETM+ and L8-OLI missions. There is an observed decrease in maximum GNDVI during 2005–2006, which continues for several years (Figure 6). However, this was not a significant impact on the GNDVI from the change detection anomaly score. Rather, there is a significant change in the winter of 2011, unrelated to the RPM infestation.

### 4.3. El Salvador Study Site

**Figure 7:**
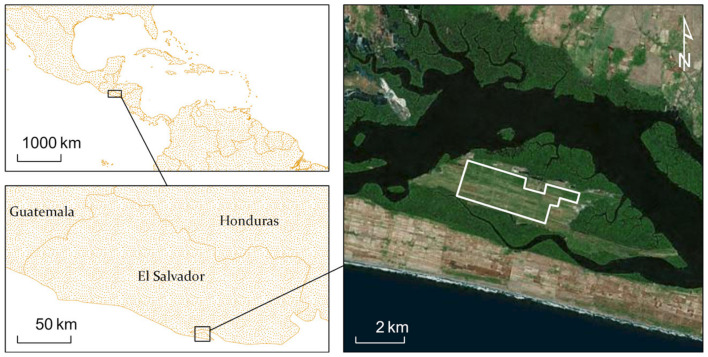
Coconut palm plantation in El Salvador along the Pacific Ocean shown in a true color Google Earth image. The area outlined in white was selected for data retrieval from Google Earth Engine.

**Figure 8:**
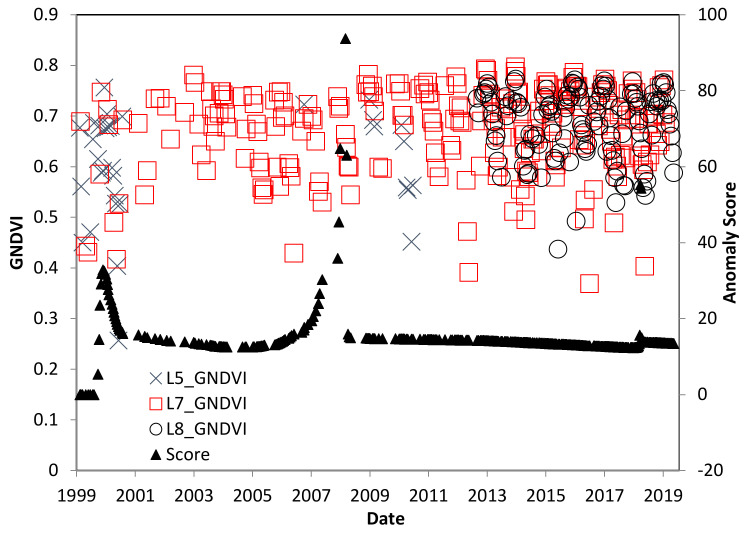
GNDVI time series for the El Salvador study site, showing variations caused by distinct rainy and dry seasons. *R. indica* was first observed at this site in 2015.

Figure 7 shows the study region in El Salvador, near the town of El Jobal on the southern coast. Significant cloud cover inhibited the retrieval of adequate assessments of GNDVI for this domain from L5-TM. L7-ETM+ and L8-OLI scenes are available for larger palm stands such as this location. GNDVI for the El Salvador study site shows a strong seasonal variation with the maximum in autumn after the rainy season and the minimum in spring after the dry season. There were no distinct or obvious deviations in either maximum or minimum GNDVI after the RPM was observed at the site (Figure 8). There is an anomaly detected in 2009, which is also unrelated to the RPM infestation, because that is known to have occurred in 2015.

### 4.4. Ceara, Brazil Study Site

**Figure 9:**
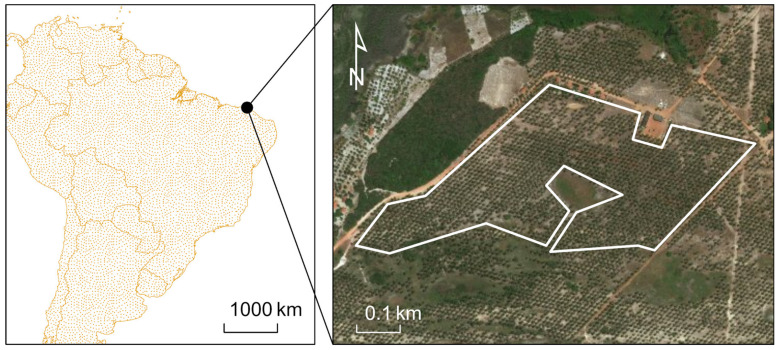
The Ceara domain in northern Brazil, with the study site highlighted in white.

The Ceara, Brazil study site is approximately 16 hectares within a very large district of approximately 5000-ha palm plantations. The smaller area was selected to reduce the influence of cloud contamination on the signal. Figure 9 shows the study region within the larger plantation.

**Figure 10:**
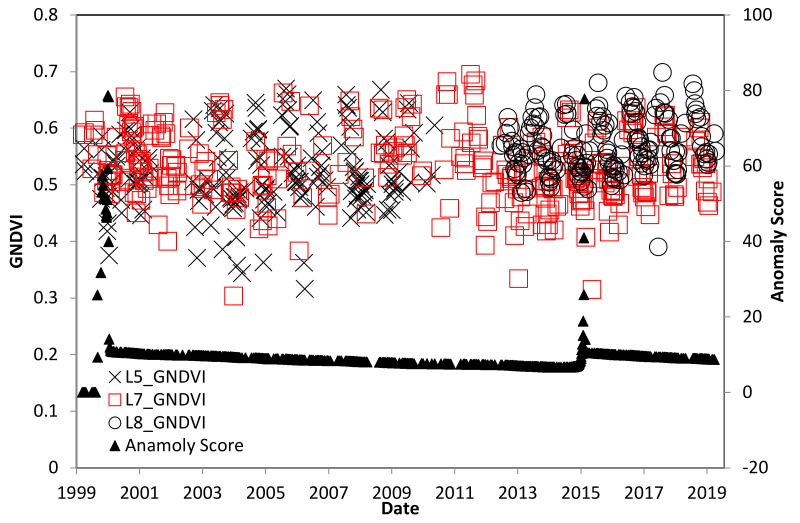
A time series of GNDVI for the Ceara Brazil study site for Landsat 5, 7, and 8.

Figure 10 shows the retrieved GNDVI values for non-cloud masked pixels within that study region. Values range from 0.3 to 0.7, which can be described as well vegetated. As these images are essentially nadir, it is likely that the yellowing palm fronds are being masked by either the healthier palm fronds at the top of the tree or increased growth of the understory. If the 2016 date for first detection of *R. indica* at this site (Table 1) occurred soon after the actual date of introduction, then there were no differences in GNDVI before and after introduction (Figure 10). However, the date of introduction may be closer to 2009, which was the year of the first record of *R. indica* in the state of Roraima, Brazil. If 2009 is closer to the actual date of introduction, then the increase in GNDVI over time may be due to the growth the understory vegetation. There is no evidence in the anomaly score that this infestation impacted the GNDVI, though there is an unrelated anomaly in November of 2015, possibly as a result of a hurricane. 

### 4.5. Pará, Brazil Study Site

**Figure 11:**
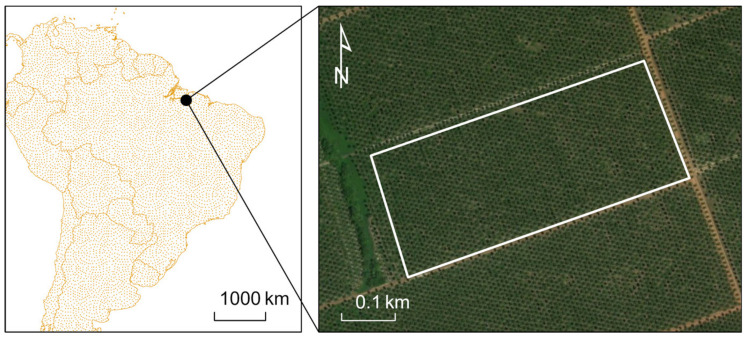
Google Earth image of the Brazil palm plantation near Para. The white outline indicates the study region.

The Para Brazilian study site is an approximately 12-hectare plot within a large 3,800-ha palm plantation. The smaller area was selected and on-ground inspection revealed absence of RPM infestation for this location. Furthermore, frequent cloud cover creates misleading statistics for larger areas within the visible and near infrared spectral ranges. Figure 11 shows the study region within the larger plantation.

**Figure 12:**
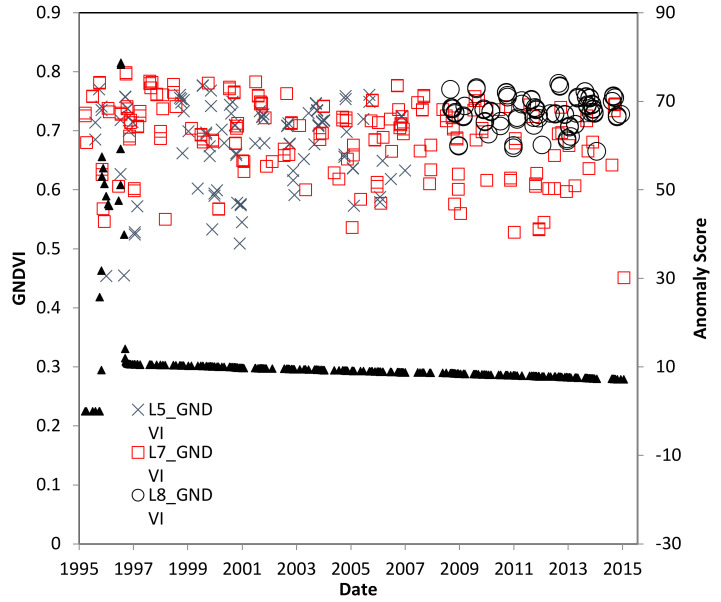
GNDVI over time for the Pará, Brazil study location. *R. indica* was not observed at this site.

Figure 12 shows the retrieved GNDVI values for non-cloud masked pixels within that study region. Values range from 0.45 to 0.85, which can be described as highly vegetated. Comparing the time series of the other time series from the study sites and there were no obvious systematic changes during this study period. In addition, there is no significant anomaly score at this site for the duration of the study.

## 5. Discussion

### 5.1. Spectral Information for Detecting Leaf Damage by RPM

The characteristic feature of a red palm mite infestation is the yellowing of the lower leaves of the palm tree, caused by a reduction of chlorophyll content. However, the lower leaves may be obscured by the upper green leaves, but given the wavelength options, green is the most useful for detection with Landsat imagery. Another possible reason could be that growth of the understory vegetation was stimulated by the extra light penetrating the canopy, so there was no change in GNDVI [55]. With smaller pixel sizes, single palms with RPM damage cover much more of the area, increasing the signal for detection.

It is not difficult to detect foliar damage that causes a reduction in leaf area index or an increase in bare ground using spectral vegetation indices. We conducted model simulations to determine if the chlorosis could lead to observable differences between RPM-infested and healthy palm plantations in a time-series of Landsat data. These simulations eliminated extensive statistical analyses of ground truth data required to determine the best bands for RPM detection.

**Figure 13:**
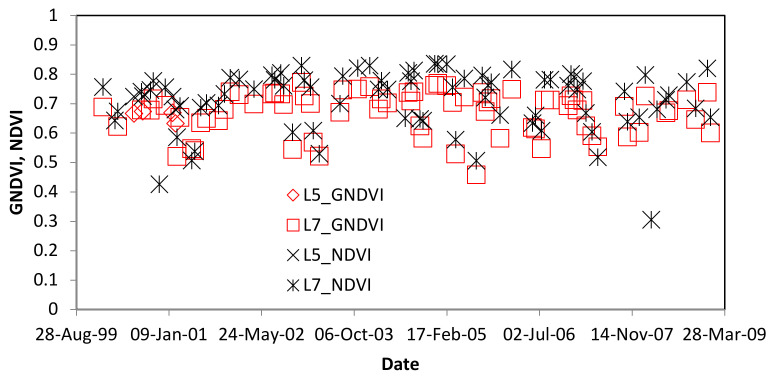
Comparison of GNDVI and NDVI for Trinidad from 2000 to 2009. First observation of RPM was in 2006.

If we had found a reduction in GNDVI at the time of RPM introduction, we would have then followed up with an NDVI analysis, because this index is less sensitive to chlorosis and more sensitive to bare ground. If there had been a significant reduction in both GNDVI and NDVI, we would have concluded there was a reduction in leaf area index and that could be linked to RPM infestation. However, if GNDVI had larger reductions than NDVI, then we would have preliminary evidence of RPM infestation. This method is analogous to detection of crop nitrogen deficiency [59,60]. Early deficiency symptoms lead to a reduction in leaf area index, affecting most vegetation indices such as NDVI and GNDVI. Later in the growing season, deficiency systems include chlorosis, affecting GNDVI and similar indices more than NDVI and related indices [60,61]. Figure 13 shows the comparison of GNDVI and NDVI for Trinidad and it is observed that there is no significant anomaly for the year 2006.

### 5.2. Spatial and Temporal Resolution

One challenge of using satellite data products to detect changes in plantation health over time is the spatial resolution of the available sensors. Over the time period of interest, Landsat satellites are the primary sensors for monitoring vegetation with visible and near-infrared bands. The resolution of these products is 30 m; therefore, many plantations will comprise just a few indistinct pixel clusters and it is difficult to identify a specific stand in an image. We overcame the challenge of locating the exact Landsat pixels in an image for all the images available in the United State Geological Survey (USGS) satellite data archive by using high-resolution images in Google Earth Engine.

Higher spatial resolution for multispectral sensor data is achieved at higher cost by pointing the satellite sensor at the desired target (e.g., Quickbird or WorldView). However, the drawback is that data collection over time becomes very expensive. High spatial resolution satellite data are most valuable when an occurrence of a problem is known, but not the problem’s severity or extent. Because Landsat data are free, the potential for monitoring for the occurrence of problems has lower cost.

Another challenge for this study was the presence of clouds. Landsat scenes are collected in the morning hours to reduce the possibility of cloud cover. However, for tropical regions there is still significant cloud cover making observation difficult. Given the temporal coverage of Landsat (16-day repeat cycle), the opportunities for coverage mean that only a few scenes may be available in a given year. In addition to Landsat 8 and planned follow-up missions, there are now additional satellites such as Sentinel-2A and Sentinel-2B [62] acquiring Landsat-like data, increasing the frequency of data collection over an area [63].

The Green Normalized Difference Vegetation Index (GNDVI) was used in this study to maximize the influence of the green channel while also reducing differences in brightness [44]. However, as shown by the El Salvador study site, there were variations in GNDVI caused by the seasonal growth cycle. There are two ways to interpret decreased vegetation indices over time. One is to assume all trees are equally and gradually damaged over time, and the other is to assume a fraction of the trees are damaged, and the remainder are healthy. The fraction of damaged palms (*f*) may be calculated from the coconut plantation’s GNDVI:

GNDVI = *f* × GNDVI_RPM_ + (1 − *f*) × GNDVI_MAX_ (3)

where GNDVI_RPM_ is the value for damaged palms and GNDVI_MAX_ is the value for healthy palms. However, GNDVI is not uniquely specific to the fraction of damaged palms. Like most vegetation indices, multiple factors and their interactions will affect the value of GNDVI_MAX_. The most important factors affecting GNDVI are the palm density and leaf area index, which also strongly affect NDVI.

### 5.3. Other Sensor Systems

This study was based on Landsat multispectral data; however, other sensors may be appropriate for different objectives.

#### 5.3.1. Off-Nadir Sensors

Sensors such those on Landsat point a few degrees off from a line pointing to the center of the Earth (nadir). The yellowing of the leaves occurs at the bottom of the tree canopy, so nadir pointing sensors are obscured by the healthy green leaves at the top of the canopy. Off-nadir imagery may be more capable of observing the visible yellowing of the leaves. As cheaper satellite platforms become available, the possibility for more non-nadir imagery is improving. In addition, for larger domains, unmanned aerial vehicles have become more feasible as a monitoring device [64].

#### 5.3.2. Synthetic Aperture Radar (SAR)

Synthetic aperture radar (SAR) would be feasible to monitor palm stand biomass because clouds are transparent to low-frequency microwaves. Japan’s Advanced Land Observation System (ALOS) Phased Array type L-band Synthetic Aperture Radar (PALSAR) collected SAR data from 2006 to 2011 showing plantation biomass could be estimated [65,66,67]. Currently, there is no SAR mission with sufficient resolution to observe the damage caused by RPM infestation. The upcoming NISAR (NASA ISRO Synthetic Aperture Radar) mission will provide field scale (about 200-m resolution L-band) microwave data (L-band is the designation for frequencies about 1.27 GHz or wavelengths of about 21 cm). Airborne instrumentation, such as the Unattended Aerial Vehicle Synthetic Aperture Radar (UAVSAR) instrument can capture high resolution L-band backscatter, but this mission is only flown when tasked, and does not provide regular flights for monitoring purposes without excessive costs.

#### 5.3.3. Light Detection and Ranging (LIDAR)

Light detection and ranging (LIDAR) uses the time between emitting a laser pulse and its return to estimate tree height and canopy volume, from which woody biomass may be estimated [68]. Current biomass missions, such as ICESAT-2 and GEDI [69], are able to identify individual tree structures using light, but there is not a sufficient historical time series to observe the red palm mite invasion in the western hemisphere. Tree-scale canopy change, such as fronds drooping when infested by RPM, is assumed to be observable via LIDAR instruments.

## 6. Conclusions

We used simulation models to predict the spectral signal expected for coconut palms in the initial stages of RPM infestation. The use of these models saves time and effort, reducing the need for extensive field work and statistical analyses. Based on the characteristic yellow leaves, reduction of GNDVI with little reduction of NDVI at the time of introduction would provide evidence of RPM infestation. However, we were not able to detect the predicted signal for a time series of L5-TM, L7-ETM+, and L8-OLI for three study sites, either from visual inspection or a change detection analysis. This may be for a variety of reasons, including the fact that the pixel sizes are too large. However, we cannot conclude that the method we developed is infeasible, because the canopy structure or the understory vegetation was stimulated as well from more sunlight due to decrease in palm canopy.

## Figures and Tables

**Table 1:** Location and characteristics of study sites. Area of study site indicates total palm orchard size in the region, and () indicates the size of the particular domain for which the statistics were calculated.

Study Site	Latitude	Longitude	Area of Study Site (hectares)	Date First RPM Observation
Trinidad	10.057° N	61.900° W	~700 (220)	October 2006
El Salvador	13.241° N	88.606° W	~1000 (500)	January 2015
Ceara, Brazil	2.974° S	39.793° W	~5000 (16)	May 2016
Pará, Brazil	2.141° S	48.630° W	~3800 (11)	None

## Appendix A

**Table A1:** Year red palm mite (*Raoiella indica* Hirst) was first detected in area.

Island/Country	Year	Reference
Martinique	2004	Flechtmann and Etienne [69]
Saint Lucia	2004	Kane et al. [70,71]
Dominica	2005	Kane et al. [70,71]
Dominican Republic	2006	Rodriguez et al. [29]
Guadeloupe	2006	Etienne and Flechtmann [72]
Saint Martin	2006	Etienne and Flechtmann [72]
Trinidad	2006	Kane and Ochoa [2]
Puerto Rico	2006	Rodrigues et al. [29]
Florida, United States	2007	Welbourn [19]; Feiber and Lemon [73]
Haiti	2007	Dowling et al. [17]
Venezuela	2007	Vásquez et al. [26]
Cuba	2007	Dowling et al. [17]
Turks & Caicos	2008	Navia et al. [74]
Mexico	2007	Beard et al. [15]
El Salvador	2015	Guzman et al. [22]
Roraima, Brazil	2009	Navia et al. [74]
Colombia	2010	Carrillo et al. [20]
Amazonas, Brazil	2010	Rodrigues and Antony [24]
Nicaragua	2016	Beard et al. [15]
Honduras	2016	Beard et al. [15]
Guatemala	2017	Garcia-Ochaeta [75]
Pará, Brazil	2018	Noronha et al. [25]
Ecuador	2020	Alcivar et al. [76]
Paraguay	2020	Ramirez et al. [77]

## Appendix B

**Table A2:** Sensor wavebands for Landsat 5 Thematic Mapper (L5-TM), Landsat 7 Enhanced Thematic Mapper Plus (L7-ETM+), and Landsat 8 Operational Land Imager (L8-OLI).

Waveband	L5-TM ^1^		L7-ETM+ ^2^		L8-OLI ^3^	
	Number	Wavelength (μm)	Number	Wavelength (μm)	Number	Wavelength (μm)
Coastal					1	0.43–0.45
Blue	1	0.45–0.52	1	0.45–0.52	2	0.45–0.51
Green	2	0.52–0.60	2	0.52–0.60	3	0.53–0.59
Red	3	0.63–0.69	3	0.63–0.69	4	0.64–0.67
Near Infrared	4	0.76–0.90	4	0.77–0.90	5	0.85–0.88
Shortwave Infrared 1	5	1.55–1.75	5	1.55–1.75	6	1.57–1.65
Shortwave Infrared 2	7 ^4^	2.08–2.35	7 ^4^	2.08–2.35	7	2.11–2.29
Panchromatic ^5^			8	0.52–0.90	8	0.50–0.68
Cirrus					9	1.36–1.38

^1^ Landsat 5: Launched 1 March 1984; Retired 5 June 2013. ^2^ Landsat 7: Launched 15 April 1999; Scanning Line Corrector malfunctioned on 31 May 2003. ^3^ Landsat 8: Launched 30 May 2013. ^4^ Band 6 was thermal infrared. ^5^ Panchromatic spatial resolution is 15 m, all other bands have 30 m resolution.
